# Kinematic and Aerodynamic Analysis of a *Coccinella septempunctata* Performing Banked Turns in Climbing Flight

**DOI:** 10.3390/biomimetics9120720

**Published:** 2024-11-22

**Authors:** Lili Yang, Zhifei Fang, Huichao Deng

**Affiliations:** Robotics Institute, Beihang University, Beijing 100191, China; lily_yang@buaa.edu.cn (L.Y.); fzf88@buaa.edu.cn (Z.F.)

**Keywords:** aerodynamic forces, banked turns, *Coccinella septempunctata*, wing kinematics, climbing flight

## Abstract

Many *Coccinella septempunctata* flights, with their precise positioning capabilities, have provided rich inspiration for designing insect-styled micro air vehicles. However, researchers have not widely studied their flight ability. In particular, research on the maneuverability of *Coccinella septempunctata* using integrated kinematics and aerodynamics is scarce. Using three orthogonally positioned high-speed cameras, we captured the *Coccinella septempunctata*’s banking turns in the climbing flight in the laboratory. We used the measured wing kinematics in a Navier–Stokes solver to compute the aerodynamic forces acting on the insects in five cycles. *Coccinella septempunctata* can rapidly climb and turn during phototaxis or avoidance of predators. During banked turning in climbing flight, the translational part of the body, and the distance flown forward and upward, is much greater than the distance flown to the right. The rotational part of the body, through banking and manipulating the amplitude of the insect flapping angle, the stroke deviation angle, and the rotation angle, actively creates the asymmetrical lift and drag coefficients of the left and right wings to generate right turns. By implementing banked turns during the climbing flight, the insect can adjust its flight path more flexibly to both change direction and maintain or increase altitude, enabling it to effectively avoid obstacles or track moving targets, thereby saving energy to a certain extent. This strategy is highly beneficial for insects flying freely in complex environments.

## 1. Introduction

The flight capability of insects is a remarkable evolutionary achievement, especially demonstrated in their precise and agile flying maneuverability. Among many flying skills, turning motion is particularly crucial. It is an indispensable ability for insects to evade predators, hunt for food, and navigate to find mates, as well as a significant indication of their adaptation to complex environments. Despite the extensive study on the turning mechanism of insects, which mainly focuses on particular species [1,2,3,4,5], research on some less common or experimentally challenging insect species remains scarce. This limitation hinders the comprehensive understanding of the diversity in insect turning behavior, leaving many unknowns in this field. This study aims to delve into the mechanism of banked turning motions during the climbing flight of *Coccinella septempunctatas*, revealing the complex biophysical processes behind it and providing new insights into biomimetics.

High-speed footage reveals that dragonflies exhibit two primary turning mechanisms in flight: the ‘conventional’ turn, which involves asymmetrical wing movements to generate a banked turn similar to an airplane and is optimal for high-speed flight, and the ‘yaw turn’, which entails rotation without banking, utilizing drag differences between wings and is suitable for low speeds or hovering [4].

Several key findings have emerged in recent research on the *Coccinella septempunctata*. The first study focused on the aerodynamic effects of the micro-structure of the beetle’s elytra during forward flight [6]. Another investigation explored the coupling structures in insects, revealing how the locking of the elytra in *Coccinella septempunctata* is a protective cover and contributes to energy absorption during falls [7]. Furthermore, research focused on the adhesion structures found on the *Coccinella septempunctata*’s feet, inspiring the development of PDMS-based adhesion structures for applications like attachment in surveillance drones or walking on inclined surfaces for small robotic devices [8]. Finally, a study investigated the rapid deployment of *Coccinella septempunctata* elytra and proposed that elastic microstructures, specifically setae on the internal edges of the elytra coupling, enable rapid opening by storing and releasing elastic strain energy [9]. These findings shed light on the biomechanics of elytra deployment and highlight the adaptability and distributed actuation pattern in *Coccinella septempunctata*. However, there is a lack of research on the kinematics and aerodynamics of *Coccinella septempunctata*s.

Researchers have achieved advancements in enhancing our understanding of the turn flight dynamics of insects. Chengyu Li and Haibo Dong found, through quantitative measurements of dragonfly wing kinematics, that there is a distinct difference in the flapping patterns between the inner and outer wings when the dragonfly performs a turning motion [3]. The analysis of fruit fly maneuvers indicated that to mitigate the motion of turning, asymmetric movements of the wings were essential [10]. Peter Windes and Danesh K Tafti reveal the asymmetry in wing kinematics and the consequent asymmetry in aerodynamic forces during turning flight by analyzing the wing kinematics data and aerodynamic simulations of the sizeable insectivorous bat (*Hipposideros armiger*) in straight and turning flight [11]. Johansson and Henningsson investigated the flight dynamics of butterflies during inclined turns by placing them in a wind tunnel and employing tomographic particle image velocimetry (Tomo-PIV) [12]. They performed a novel analysis to study the aerodynamics and kinematics associated with banked take-off turns [13]. There is currently no research on the banked turns of beetles during climbing flight, which is a topic worth studying.

By studying the dynamic characteristics of wing flapping and the crucial adjustments of body posture for flight control, this research investigates the coupling effects of three parameters—flapping angle, stroke deviation angle, and rotation angle—in the *Coccinella septempunctata* beetle. Additionally, it examines how this coupling enables the beetle to perform complex flight maneuvers. Through detailed experimental observations and theoretical analysis, we have found that the distance covered by upward and forward flight during inclined turns is greater than that of rightward flight. During inclined turning, the beetle achieves smooth climbing and turning by controlling its body roll angle in the first four cycles, followed by a 40° rotation in the latter half of the cycle. The *Coccinella septempunctata* beetle exhibits remarkable agility and stability. This finding highlights the effectiveness and precision of the beetle in spatial orientation and navigation, with potential implications for guiding micro-aircraft in long-range missions.

## 2. Materials and Methods

### 2.1. Biological Experiment

The study focused on *Coccinella septempunctata* during their free-flight maneuvers. *Coccinella septempunctata* were captured in the school garden. The flight experiments were conducted between 12:00 and 14:00 on the capture day when insect activity was at its peak. To ensure that the experiments would be successful, the researchers selected only the most vigorous subjects. The *Coccinella septempunctata* primarily exhibits climbing flight because of its positive phototactic behavior. There was a vertical branch positioned in the center of the flight zone. The *Coccinella septempunctata* take off from the top of this branch, and the data are collected during the stable phase of free flight.

The body and wing kinematics of *Coccinella septempunctata* in free flight were quantified using three synchronized high-speed cameras (i-SPEED 716, iX Cameras, Essex, UK) that were arranged orthogonally on an optical table. To meet the requirements for accuracy in both time and space, the cameras were configured to capture at 5000 frames per second (with a resolution of 2048 × 1536 pixels) and a shutter speed of 20 μs. As a result, each wingbeat cycle consisted of approximately 70 images. Each of the cameras was fitted with a 60 mm micro-Nikkor lens to meet the requirements of the filming area, which is about 1.5 × 1.5 × 1.5 cm^3^. The camera view was illuminated from behind with a 215 W integrated light-emitting diode (LED), providing a luminous flux of 95,600 lm, supporting precise brightness adjustment from 0% to 100%, and operating at a wavelength of 780 nm. The synchronized cameras were operated manually when the insect was noticed to be flying steadily within the recording space. The ambient temperature was maintained between 25 °C and 27 °C, while the relative humidity was kept at 50% to 60%.

**Figure 1:**
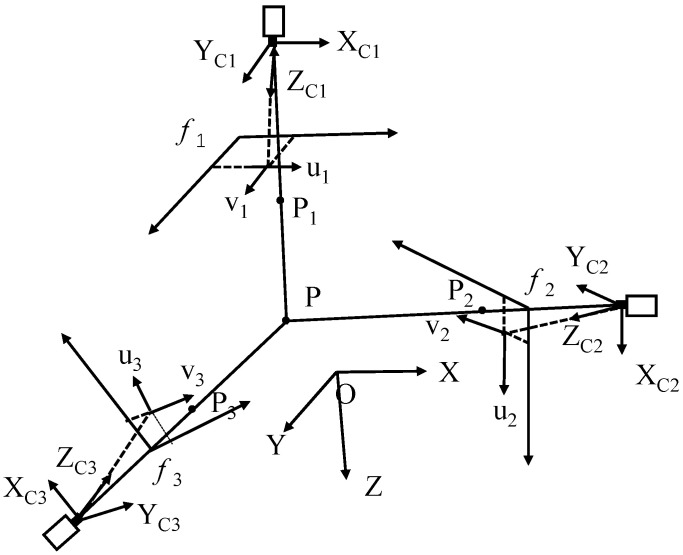
Trinocular Stereo Vision System Model.

By utilizing a flat calibration panel to calibrate the cameras, it is possible to derive the transformation matrices that relate the world coordinate system to each of the three image coordinate systems, known as projection matrices. According to the foundational principles of stereo vision, if the coordinates of a point in the world coordinate system and the corresponding projection matrices are known, one can compute the projections of that scene point onto any image coordinate system [14]. Figure 1 depicts the overall configuration of a trinocular stereo vision system. (*X*, *Y*, *Z*) represents the global coordinate system, while (*X_C_*_1_, *Y_C_*_1_, *Z_C_*_1_), (*X_C_*_2_, *Y_C_*_2_, *Z_C_*_2_), and (*X_C_*_3_, *Y_C_*_3_, *Z_C_*_3_) denote the coordinate systems for cameras 1, 2, and 3, respectively. The image coordinate systems for these cameras are given by (*u*_1_, *v*_1_), (*u*_2_, *v*_2_), and (*u*_3_, *v*_3_). *P* represents an arbitrary point in space, while *p*_1_, *p*_2_, and *p*_3_ are the corresponding projective points. Additionally, *f*_1_, *f*_2_, and *f*_3_ refer to the focal lengths of cameras 1, 2, and 3, respectively.

In this study, we focused on observing the most active individual of *Coccinella septempunctata* by collecting approximately 5 separate recordings of the same free-flying *Coccinella septempunctata* using a calibrated high-speed video apparatus during its peak activity time, between 12 PM and 2 PM on the same day. Due to its phototactic nature, the *Coccinella septempunctata* exhibited similar movement patterns across all five trials. These recordings were carefully examined, and one recording was selected for further study; in the selected recording, the *Coccinella septempunctata* was performing a turning maneuver with similar flight characteristics, and the image acquisition quality was high quality for all three cameras. For this research, during right turns, the left wings are referred to as the outer wings, and the right wings as the inner wings.

**Figure 2:**
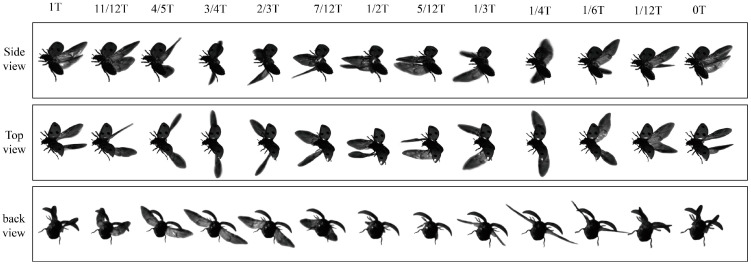
Videos of *Coccinella septempunctata* in climbing motion, presented from the perspectives of three cameras. The time notations are non-dimensionalized for the cycle.

In this study, the free-climbing flight of *Coccinella septempunctata* was successfully recorded. The video sequences in Figure 2 (multimedia view) illustrate a complete flight cycle. At *t* = 0, the sequences show the initiation of the downstroke in the flight of *Coccinella septempunctata*.

**Figure 3:**
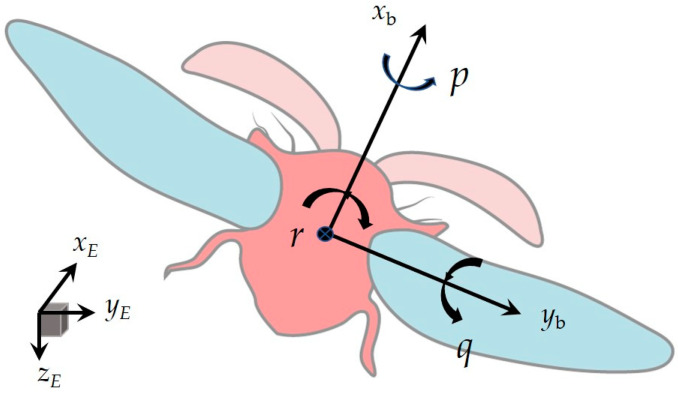
Reference coordinate system (*x*_E_, *y_E_*, *z_E_*) and the body angular velocity components along the three axes of the body-fixed frame (*x_b_*, *y_b_*, *z_b_*): *p* (roll rate), *q* (pitch rate), *r* (yaw rate).

We use Etkin and Reid’s flight dynamics convention [15] to describe body kinematics. We establish two frames of reference in Figure 3: the Earth-fixed frame (*x*_E_, *y*_E_, *z*_E_) with horizontal *x*_E_ and *y*_E_ axes and a vertical *z*_E_ axis that points downward, and the insect’s body-fixed frame (*x*_b_, *y*_b_, *z*_b_) with its origin at the center of mass, the *x*_b_ axis running along the insect’s body from tail to head, and the *y*_b_ axis pointing to the insect’s right side. In the present study, we specify the orientation of the insect’s body using three Euler angles, *ψ*, *θ*, *ϕ*, which we refer to as the body’s heading, elevation, and bank angles, respectively [15]. When analyzing the movements that produce the maneuver, using the components of the body’s angular velocity along the three axes of the body-fixed frame (*x*_b_, *y*_b_, *z*_b_) proves convenient. We denote them as *p*, *q*, and *r* for the roll, pitch, and yaw rates in Figure 2. We refer to *x*_b_, *y*_b_, and *z*_b_ as the roll, pitch, and yaw axes, respectively, with *x*_b_ and *y*_b_ also known as the body’s long and transverse axes. The following Equation (1) relates *p*, *q*, and *r* to the Euler angle rates:

(1) $$\left[\begin{array}{c} p\\ q\\ r \end{array}\right]=\left[\begin{array}{ccc} 1 & 0 & -\sin\theta \\ 0 & \cos\phi & \sin\phi \cos\theta \\ 0 & -\sin\phi & \cos\phi \cos\theta \end{array}\right]\left[\begin{array}{c} \dot{\phi }\\ \dot{\theta }\\ \dot{\psi } \end{array}\right]$$

**Figure 4:**
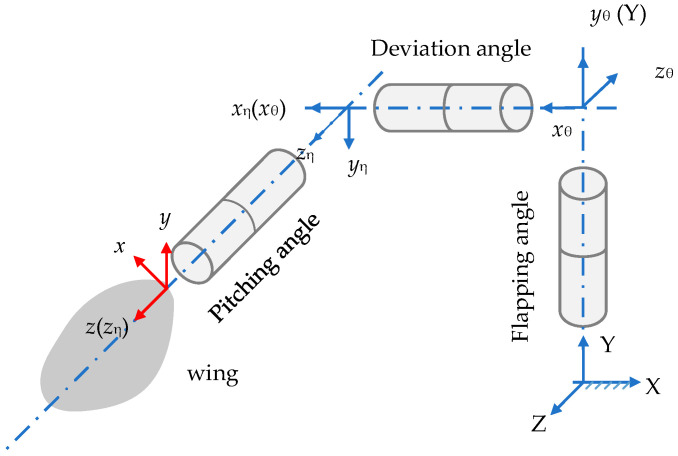
Wing kinematics parameters and coordinates.

Because the wings connect to the body through the wing roots and follow the body’s movement, the wings maintain a fixed position relative to the body. To describe the flapping motion of the wings, we adopt Ellington’s method to establish a coordinate system for wing flapping in a plane (the stroke plane) at the wing roots, which moves following the body [16]. Figure 4 defines the flapping angle (*ϕ*_w_), the deviation angle (*θ*_w_), and the pitching angle (*α*_w_) of a wing as illustrated. The kinematics of a rigid flapping wing is described through successive rotations, utilizing the ‘cans in series’ method introduced by Schwab and Meijaard [17]. This analysis involves four distinct frames: the inertial frame *XYZ*, two intermediate frames *x_θ_y_θ_z_θ_* and *x_η_y_η_z_η_*, as well as the co-rotating frame *xyz*. Although these frames are represented in different positions, they all originate from the same point.

After documenting the flight, we administered anesthesia to the *Coccinella septempunctata* and subsequently determined its overall mass using a laboratory scale (Sartorius AG BT25S) sourced from Göettingen, Germany. This particular scale boasts an impressive precision level of up to ±0.01 mg. Subsequently, the wings were removed from the body, and the mass of the wingless body was measured. The wing mass (*m*_wg_) was determined by subtracting the mass of the wingless body from the total mass. We digitally captured the wings’ configuration with a microscope equipped with an electronic eyepiece with a display resolution of 2048 × 1536. A specialized toolbox was developed for MATLAB R2020a (The MathWorks, Inc., Natick, MA, USA) to accurately extract the position and orientation of both the body and wings from images acquired by all three cameras. A line segment, equal in length to the body of the insect, was taken to represent the insect’s body, and the outline of the wing obtained from a scanned image was used to represent the wing [14]. The body’s line segment and the wing’s outline are referred to as the respective models for the body and wing. The positions and orientations of those models were adjusted until the optimal alignment between a model’s projection and the displayed frame was achieved in all three views. The achievement of a satisfactory overlap often necessitated multiple adjustments in both the position and orientations of each model.

The approach used to measure the morphological parameters was identical to that described by Mou et al. [18]. The wing length refers to the length of the segment connecting the wing root and the wingtip. The single wing area can be measured from the scanned image of the wing profile. The definitions of the average chord length *c*, aspect ratio *AR*, and the second moment of area of the wing *r*_2_ are as follows:

(2) c=S/R

(3) $$AR=2R^{2}/S$$

(4) $$r_{2}^{2}=\frac{1}{S}\int_{S}r^{2}dS$$

Table 1 provides the morphological parameters and kinematics parameters of the *Coccinella septempunctata*, including the total mass of an insect (*m*), single wing mass (*m*_w_), wing length (*R*), average wing chord length (*c*), aspect ratio *AR*, wing area (*S*), the radius of the wing area’s second moment (*r*_2_), mean tip velocity of the hind wing *U*_h_, sweeping amplitude *Φ*, and frequency *f*.

### 2.2. Classification of Turning Flight

To compare the turning angles of the five phases of a *Coccinella septempunctata*, the body movement is first transformed from a body-fixed coordinate system to a global coordinate system to establish their tangential velocity vectors. The three-dimensional positions are projected onto the plane parallel to the horizontal plane (the *X_E_Y_E_* plane of the global coordinate system) to categorize the turning angles. However, the turning trajectories of the *Coccinella septempunctata* are not circular arcs but deviate in an *S*-shape, making it difficult to directly estimate the turning radius. In this study, the least squares method (LSM) is utilized to calculate the fitted circle and the corresponding radius based on the trajectory data. The study also assumes that *Coccinella septempunctata*s maintain similar body postures and wing movements when flying, with a similar turning radius [19].

The most commonly used least squares fitting method for a circle, Kasa [20], is a simple and fast method for estimating the center position of a circle. The general equation for a circle can be expanded into Formula (2). Since the trajectories of the *Coccinella septempunctata*s in flight are projected onto the ground, the data points on the plane are scattered. The distance from each point to the center of the circle is denoted as *h_i_*; *X_i_* and *Y_i_* represent the coordinates of a point on the trajectory; and the error between this distance and the circle radius is denoted as *e_i_*. The sum of the squares of all errors is expressed as *f*(*a*, *b*, *c*), as shown in Formula (4). By taking the partial derivatives of the objective function *f*(*a*, *b*, *c*) concerning the coefficients *a*, *b*, and *c* and setting them to zero, three linear equations can be obtained to find the minimum value of *f*. Once the coefficients *a*, *b*, and *c* are determined, they can be substituted into Equation (2) to obtain the center position and radius of the best-fit circle.

(5) $$x^{2}+y^{2}+ax+by+c=0$$

(6) $$e_{i}=h_{i}^{2}-R^{2}=X_{i}^{2}+Y_{i}^{2}+aX_{i}+bY_{i}+c$$

(7) $$f\left(a,b,c\right)=\sum e_{i}^{2}={\sum \left(X_{i}^{2}+Y_{i}^{2}+X_{i}+Y_{i}+c\right)}^{2}$$

One advantage of using the Kasa method is that if the point (*x_i_*, *y_i_*) lies exactly on the circle, Kasa can find the precise circle. However, when the sample points are only located on a small circular arc, Kasa may deviate significantly, estimating a smaller circle. Thus, the method may underestimate the radius of the fitted circle when the arc is shorter than a full circular trajectory. Additionally, the irregular flight path of a *Coccinella septempunctata* can lead to significant errors in estimating the circle’s radius. The method proposed by Pratt [21] is similar to the Kasa method, but Pratt defined the general equation of the circle differently by adding a coefficient to the quadratic term, expressed as:

(8) $$a\left(x^{2}+y^{2}\right)+bx+cy+d=0$$

(9) $$g(a,b,c,d)=\frac{\sum e_{i}^{2}}{{\left(2R\right)}^{2}}=\frac{\sum {\left[{\left(X_{i}-A\right)}^{2}+{\left(Y_{i}-B\right)}^{2}-R^{2}\right]}^{2}}{{\left(2R\right)}^{2}}$$

Pratt’s circle fitting method uses the constraint equation *b*^2^ + *c*^2^ − 4*ad* = 1 to compute the partial derivatives of the objective function *g*(*a*, *b*, *c*, *d*) concerning the coefficients *a*, *b*, *c*, *d*. By setting the partial derivatives to 0, the minimum of the objective function can be obtained, allowing for a more accurate estimation of the best-fit circle. Therefore, Pratt’s circle fitting method is chosen as the classification method for turning trajectories, with parameters as shown in Table 2.

**Figure 5:**
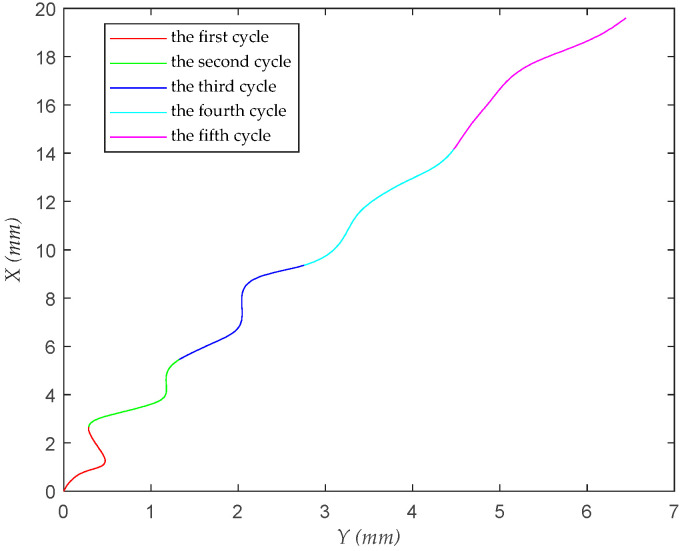
Turning radius of a *Coccinella septempunctata* on the *XY* plane.

Pratt’s method has two distinct advantages. One is that it eliminates the influence of the radius, allowing the algorithm to function correctly even when the curvature radius is very large and the noise is high. The other is that it integrates the line equation into its formulation; when the coefficient *A* = 0, the resulting curve is a straight line. This is very effective in practical applications where it is unclear whether the curve to be fitted is a line or a curve. Additionally, the Pratt method is quite efficient, making it a very practical approach. Figure 5 indicates that the *Coccinella septempunctata* ‘s flight trajectory forms a distinct *S*-shape.

### 2.3. Numerical Methods and Simulation Setup

We used the commercial software ANSYS Fluent 2021 to conduct a numerical simulation study of the banked turn in the climbing flight of *Coccinella septempunctata*. Fluent employs the multigrid method for computations, discretizing the computational domain into small grid cells and integrating the governing equations within each cell, ultimately obtaining the numerical solution for the entire computational domain. When simulating the wings of *Coccinella septempunctata*, a pressure outlet boundary condition is employed. The simulation involves the use of overlapping mesh and dynamic mesh techniques to model the wing motion of real insects, with wing movement data implemented through User-Defined Functions (UDFs). The pressure–velocity coupling is achieved using the coupled scheme, while the gradient is computed using the least squares cell-based method. The pressure and momentum discretization are carried out with second-order accuracy, using the second-order and second-order upwind schemes, respectively. Turbulent kinetic energy is also discretized using the second-order upwind approach, as is the specific dissipation rate. The time discretization is implemented using a first-order implicit scheme. The three-dimensional unsteady incompressible Navier–Stokes (N–S) fluid dynamics equations under an inertial Cartesian coordinate system during the banked turns maneuver phases are as follows:

(10) ∇⋅u=0

(11) $$\frac{\partial u}{\partial t}+u\cdot \nabla u=-\nabla p+\left(\frac{1}{Re}\right)\cdot \nabla^{2}u$$

where **u** represents the velocity vector of the fluid, *p* corresponds to the fluid pressure, and *t* is the time, ∇ functions as the gradient operator, $\nabla^{2}$ as the Laplacian operator, and *Re* represents the Reynolds number.

(12) U=2ΦfR

(13) $$Re=\frac{Uc}{\nu }$$

The reference length and velocity are chosen based on the wing’s mean chord length, denoted as *c*, and the mean flapping velocity at the wing’s tip, represented by *U*, respectively. Consequently, for the insect considered in this study, the Reynolds number *Re* is calculated to be 1234. The computation uses a model wing, sets its section thickness at 3% of the local chord length, and rounds its leading and trailing edges. We chose 3% of the mean chord length as the thickness of the wing model because previous studies have shown that thickness values within the range of 1–5% have negligible impact on the aerodynamics of the wing. Therefore, we opted for an intermediate value of 3% to balance the trade-off between structural integrity and aerodynamic performance [22,23].

**Figure 6:**
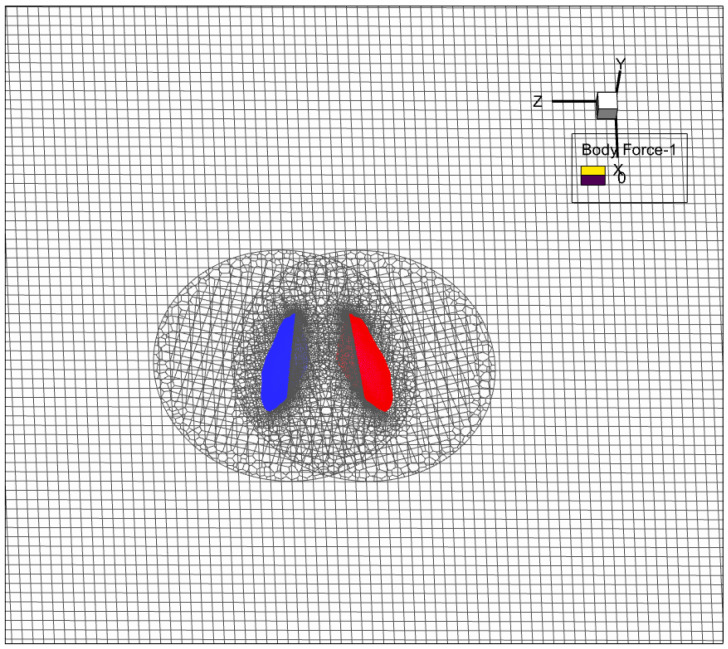
Portions of a computational grid system.

The model’s wings feature a design of flat plates with subtly curved edges at the front and back, with the plate’s size expanding to a thickness of 0.03 *c*. The movement between the left and right rear wings necessitates employing overlapping moving grids for equation solving. Each wing is assigned a body-aligned curvilinear grid, coupled with a Cartesian grid encompassing the flow domain’s distant far-field boundary, as shown in Figure 6.

**Figure 7:**
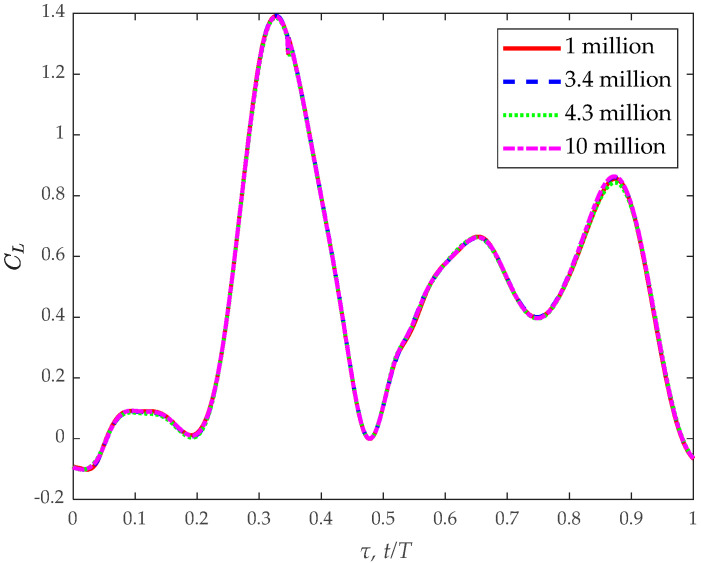
Illustrates the temporal evolution of the lift coefficient (CL) during the banked turn in the climbing flight for *Coccinella septempunctata* across different grid numbers within one cycle.

Before investigating the dynamics of flows and aerodynamic forces, we conducted a grid resolution test. We evaluated four different grids: one consisting of 1 million cells, another with 3.4 million cells, a third with 4.3 million cells, and a fourth with 10 million cells. Utilizing these grids, we performed calculations for the climbing flight of a *Coccinella septempunctata*; Figure 7 shows that the variation in lift coefficient between insect flights remains within a negligible margin as the grid size increases by increments. A more refined grid facilitates the generation of an accurate flow field; therefore, our preference lies in employing the grid containing 4.3 million cells.

## 3. Results and Discussion

During the flight, a *Coccinella septempunctata* can simultaneously rotate around its longitudinal lateral axis and dorsoventral axes of the body to turn. Measuring wing position against a fixed external horizon could be misleading, as the *Coccinella septempunctata*’s rolling motion would increase the wing stroke angle on one side and decrease it on the other. Due to the phototactic behavior exhibited by *Coccinella septempunctata*, most initiate their flight by executing a right turn toward the sunlight. During this maneuver, we designate the insect’s left wing as the “outer wing” while referring to the right wing as the “inner wing” since we analyze right turns exclusively.

### Body and Wing Kinematics of the Banked Turn in Climbing Flight

The three independent Euler angles are among the most frequently used and widely acknowledged parameters in describing reference orientations. Within this section, we define these angles and formulate the transformation matrix based on them. Euler angles encompass three consecutive rotations around axes that are not generally orthogonal. It is worth noting that Euler angles lack uniqueness; hence, we adhere to the extensively employed set proposed by Goldstein. To achieve this, we execute a sequence of three successive rotations, known as Euler angles, to carry out the transformation between two coordinate systems.

(14) $$A=\left[\begin{array}{ccc} \cos\psi_{w}\cos\phi_{w}-\cos\theta_{w}\sin\phi_{w}\sin\psi_{w} & -\sin\psi_{w}\cos\phi_{w}-\cos\theta_{w}\sin\phi_{w}\cos\psi_{w} & \sin\theta_{w}\sin\phi_{w}\\ \cos\psi_{w}\sin\phi_{w}+\cos\theta_{w}\cos\phi_{w}\sin\psi_{w} & -\sin\psi_{w}\sin\phi_{w}+\cos\theta_{w}\cos\phi_{w}\cos\psi_{w} & -\sin\theta_{w}\cos\phi_{w}\\ \sin\theta_{w}\sin\psi_{w} & \sin\theta_{w}\cos\psi_{w} & \cos\theta_{w} \end{array}\right]$$

The angles for flapping (*ϕ*_w_), stroke deviation (*θ*_w_), and wing rotation (*α*_w_) are delineated as illustrated in Figure 4. The geometric angle of attack for a wing, denoted as α, correlates with αw such that *α* = *α*_w_ during the downstroke, and *α* = 180° − *α*_w_ during the upstroke. The angle *η*, representing the angle between the stroke plane and the body’s long axis, is defined as 61.01°.

**Figure 8:**
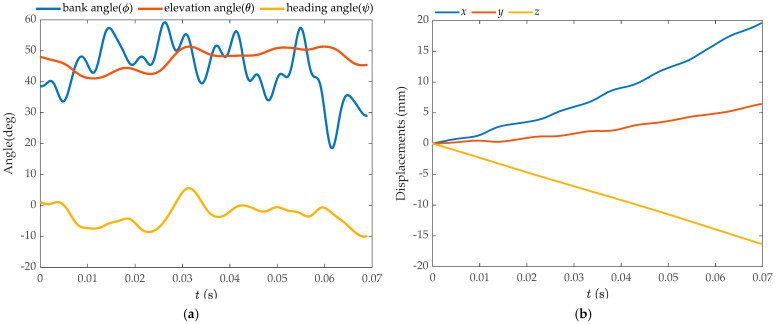
Variations in Euler angles (**a**) and center of mass displacement (**b**) of a *Coccinella septempunctata* during a banking turn.

**Figure 9:**
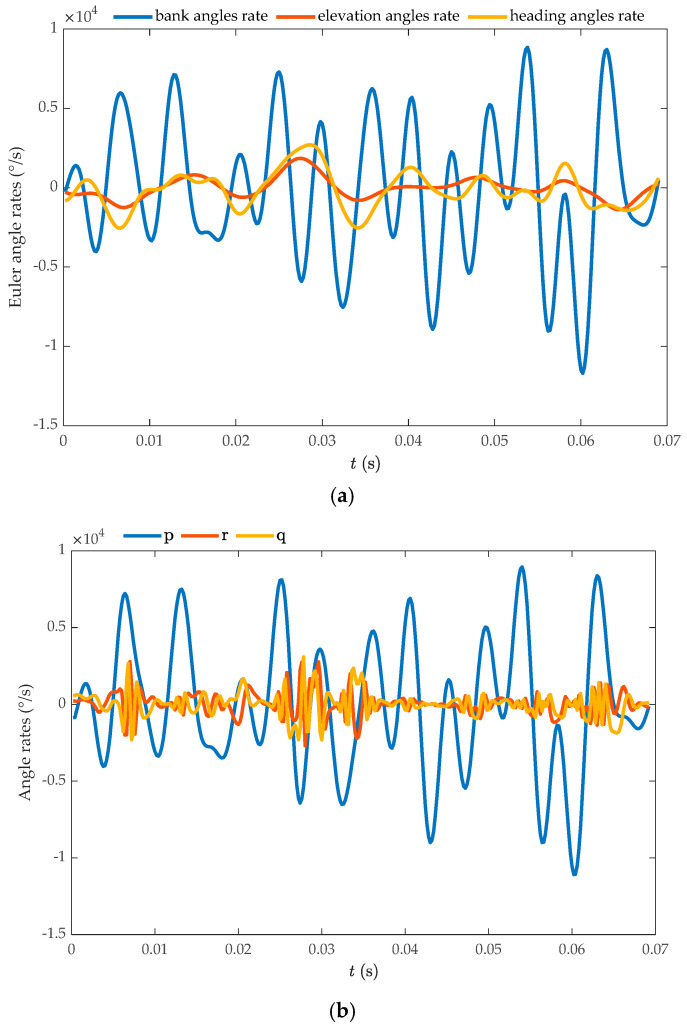

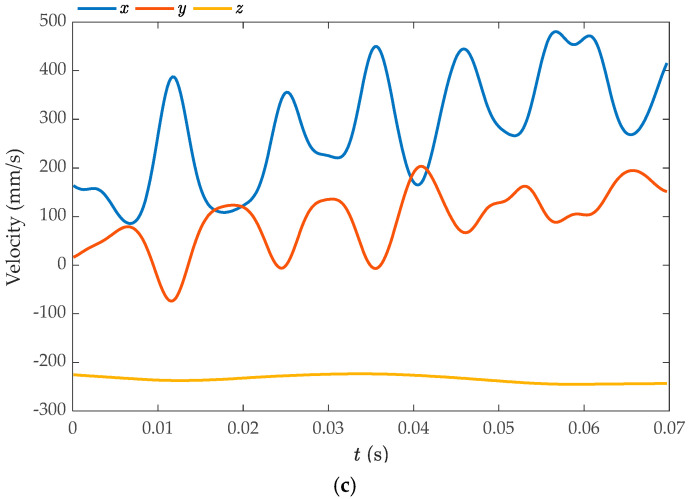
Temporal records of the movements of the *Coccinella septempunctata*’s body, illustrated as the (**a**) rates of Euler angles; (**b**) rates of roll, pitch, and yaw movements; and (**c**) movement speed of the body’s center of mass, detailing *u*_c_, *v*_c_, and *w*_c_ for the translational velocity components and *x*_E_, *y*_E_, *z*_E_ for the spatial velocity components of the body’s mass center.

Figure 8 displays the time-dependent measurements of the body’s Euler angles (*ψ*, *θ*, *ϕ*), the body’s center of mass displacements, and *Coccinella septempunctata* executing a banked turn. Figure 8a reveals that the *Coccinella septempunctata* performs banked turns during its climbing flight over five cycles (*T* = 0.0138 s). The rolling motion along the body’s long axis plays a significant role. During *t* = 0–0.055 s, the body roll angle continuously adjusts the flight posture by performing left and right rolls. During *t* = 0–0.035 s, there is a coupling effect of the three parameters: the flapping angle, the deviation angle, and the pitching angle. During *t* = 0.055–0.062 s, which is half a cycle, the insect’s body roll angle changes by 40°. Figure 8b shows that during the climbing tilted turn, the seven-spotted *Coccinella septempunctata* beetle travels a longer distance forward and upward than in rightward flight. This flight pattern is well suited for turning flights at high altitudes. We compute the Euler angle rates $\dot{\psi },\dot{\theta },\dot{\phi }$ for *Coccinella septempunctata* using the data *ψ*, *θ* and *ϕ*, and display the results in Figure 9a. The bank angle rate $\dot{\phi }$ and roll rate p is large (maximum magnitude of $\dot{\phi }$ is around 11,700 °/s and maximum magnitude of p is around −11,073 °/s), whilst the heading and elevation rates ($\dot{\psi },\dot{\theta }$) are relatively small. In the turning motion of *Coccinella septempunctata*, the bank angle plays a major role as shown in Figure 9a,b.

The rotation rates around the body-fixed *x*_b_, *y*_b_, and *z*_b_ axes, namely *p*, *q*, and *r*, are calculated using Equation (1) based on the data $\dot{\psi },\dot{\theta },\dot{\phi }$. Figure 9b displays these estimated rates. Utilizing the data on the displacement of the body’s center of mass, we calculated the translation velocity and presented it in Figure 9c. The *Coccinella septempunctata* ascends vertically at a constant speed and exhibits an acceleration-deceleration movement in the forward and rightward directions.

**Figure 10:**
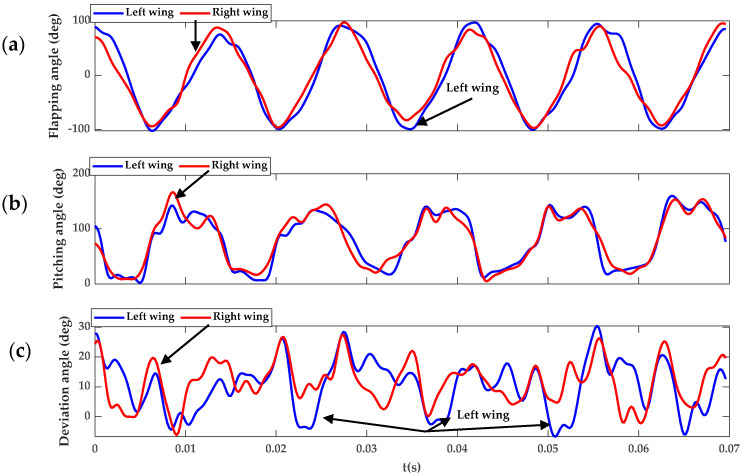
Instantaneous wing kinematics of *Coccinella septempunctata*, (**a**) *ϕ*_w_, the flapping angle; (**b**) *θ*_w_, the deviation angle; and (**c**) *α*_w,_ the pitching angle.

**Figure 11:**
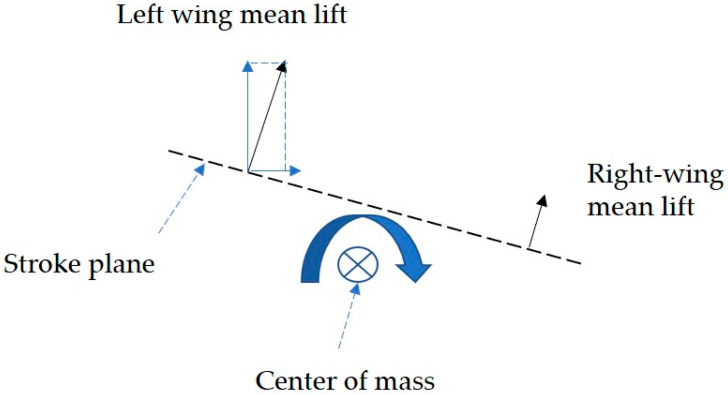
Diagram illustrating insects performing inclined turns at the center of mass.

Figure 10 shows that during the banked turn in the climbing flight, the flapping angle (*ϕ*_w_) and the deviation angle (*θ*_w_) vary significantly between the left and right wings, while the differences in the rotation angle *α*_w_ across the wings remain relatively minor. During the climbing flight, the *Coccinella septempunctata* performs banked turning maneuvers, resulting from the combined effects of wing flapping angle, stroke deviation angle, and rotation angle in insects. The data from the insect wing kinematics elucidate the banked turning in the climbing flight depicted in Figure 8. The asymmetry of insect wings is the reason behind *Coccinella septempunctata*s’ ability to climb and turn. The force generated by the left wing increases with the amplitude of its flapping angle and the increase in the lift and angle of attack. As the *Coccinella septempunctata* climbs to the right, it performs a banked turn. This banked turn generates a centripetal force towards the right, allowing for the execution of the turn. Additionally, it generates a vertical force, which is beneficial for the climbing flight, as shown in Figure 11.

**Figure 12:**
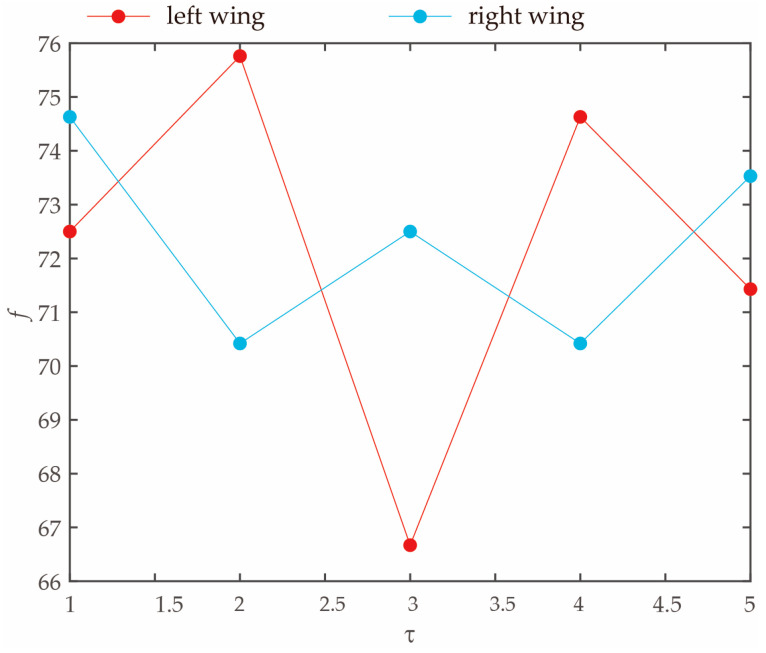
Diagram illustrating insects performing inclined turns at the center of mass.

As discussed in Figure 12, it can be observed that during the climbing flight of the *Coccinella septempunctata*, there is an asymmetry in the frequencies of the left and right wings in five cycles. This asymmetry is conducive in achieving turning flight in insects and maintaining stability. Insects typically achieve turning movements by adjusting the asymmetry in the flapping frequency of their wings. Specifically, when the flapping frequency on one side increases while it decreases on the other side, it results in an imbalance in the lift and thrust, causing the insect to turn towards the side with the higher flapping frequency.

**Figure 13:**
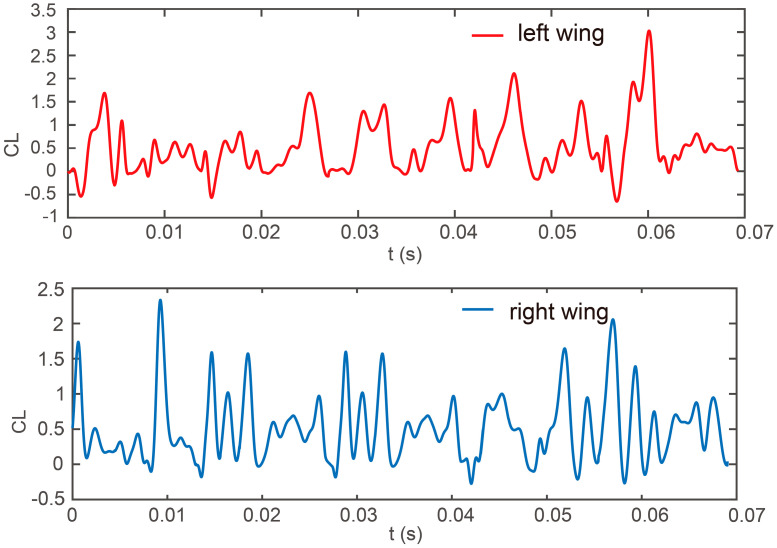
The progression of the coefficients for wing lift and F force over five cycles.

Figure 13 depicts the time courses of the lift coefficients on the left and right wings in five cycles. In Figure 13, the *Coccinella septempunctata*’s left- and right-wing lift coefficients are asymmetric and continuously changing. Within five cycles, there is no situation where one wing is consistently more significant than the other wing. During the climbing flight and inclined turning, the *Coccinella septempunctata* continuously adjusts its altitude to maintain stability. At times *t* = 0.01 s and *t* = 0.06 s, there is an increase in the lift coefficient of the right wing compared to the left wing, resulting in turning to the left and right.

From Figure 10, it can be observed that during the time interval from *t* = 0.055 s to 0.062 s, which represents half a cycle, the flapping angle, deviation angle, and pitch angle of the left wing are greater than those of the right wing, resulting in the left wing generating more lift than the right wing, as shown in Figure 13. The insect’s body is inclined at an angle of 45° to the right, exhibiting a slight yaw to the right and upward lift. This asymmetrical motion of the left and right wings facilitates the turning motion of the insect.

## 4. Conclusions and Future Work

The study presented in this paper focuses on the inclined turning of the *Coccinella septempunctata* during the climbing flight. The body’s center of mass travels farther forward and upward than to the right during flight. The *Coccinella septempunctata* ascends vertically at a constant speed and exhibits an acceleration-deceleration movement in the forward and rightward directions. Investigating the coupling effect of the three body attitude angles concerning the banked turning of the *Coccinella septempunctata*, Figure 8a illustrates the utilization of the primary body roll angle in achieving banked turning. During *t* = 0–0.0234 s, the insect’s body exhibits a left yaw deviation of approximately 10°and a decrease of around 10° in the pitch angle. During *t* = 0.055–0.062 s, corresponding to half a period, the insect undergoes a 40° turn.

From Figure 8a and Figure 10, it can be seen that the flapping angle of the *Coccinella septempunctata* influences the turning primarily by affecting the body’s yaw direction. The variations in pitch angle are relatively small for the left and right wings of the *Coccinella septempunctata*. As can be seen from Figure 10, the deviation angle is positive, making the *Coccinella septempunctata* fly upward in the direction of gravity. Through Figure 8a and Figure 10, it can be seen that the asymmetry of the left and right deviation angle has an impact on turning. From Figure 10, it can be observed that during the time interval from t = 0.055 s to 0.062 s, which represents half a cycle, the flapping angle, deviation angle, and pitch angle of the left wing are greater than those of the right wing, resulting in the left wing generating more lift than the right wing, as shown in Figure 13.

The *Coccinella septempunctata* demonstrated its phototactic behavior in the experiment and exhibited a flight state of climbing that was inclined to turn. By analyzing the kinematic and aerodynamic data from five cycles, we can observe that during the first four cycles, the beetle mainly moves upward and forward while smoothly adjusting its body roll angle to execute turns. Moreover, it can achieve a 40° turn within the last half of a cycle. These findings have instructive implications for developing flapping-wing aircrafts with long-range objectives.

In future work, the authors aim to incorporate a simulation of the interactions between the wings to further elucidate the impact of the clap-fling mechanism on the climbing flight and banked turning in *Coccinella septempunctata*, building upon their previous research on this species. Furthermore, it would be interesting to investigate whether the elytra play a specific role in inclined turning, which differs from the commonly accepted view of their protective function.

## Figures and Tables

**Table 1:** Characteristics of both morphology and kinematics in *Coccinella septempunctata*.

Parameters	Hind Wing
Flapping amplitude (*Φ*)	190°
Mean chord length ($\bar{c}$)	3.5 mm
Wing length (*R*)	11.4 mm
Aspect ratio (*AR* = *R*/$\bar{c}$)	3.257
*r*_2_	6.2027 mm
Mass (*m*)	0.00032 g
Frequency (*f*)	72.5
Total mass (including legs)	0.0297 g
Mean tip velocity of the hind wing (*U_h_*)	5.287 m/s

**Table 2:** Parameters related to the turning radius equation for five cycles.

Period/Parameter	*a*	*b*	*c*	*d*	*A*	*B*	*R*	*R* ^2^	Adjusted *R*^2^
First cycle	0.2287	−0.7183	0.7943	0.1604	1.5705	−1.7366	2.1865	0.4171	0.3997
Second cycle	0.2258	−2.1316	0.4293	4.1274	4.7199	−0.9506	2.2142	0.7760	0.7694
Third cycle	0.3551	−5.3815	−1.0744	20.4986	7.5777	1.5129	1.4081	0.8590	0.8548
Fourth cycle	0.0520	−0.9011	−1.3065	7.3002	8.6623	12.5595	9.6134	0.9666	0.9657
Fifth cycle	0.0559	−1.5334	−1.5092	16.4931	13.8841	13.4890	8.9381	0.9422	0.9405

## Data Availability

The datasets used and analyzed during the current study are available from the corresponding authors upon reasonable request.
